# In Vitro Selection of an ATP-Binding TNA Aptamer

**DOI:** 10.3390/molecules25184194

**Published:** 2020-09-13

**Authors:** Li Zhang, John C. Chaput

**Affiliations:** 1 Departments of Pharmaceutical Sciences, University of California, Irvine, CA 92697-3958, USA; zltc1206@hotmail.com; 2 Department of Chemistry, University of California, Irvine, CA 92697-3958, USA; 3 Department of Molecular Biology and Biochemistry, University of California, Irvine, CA 92697-3958, USA

**Keywords:** aptamer, TNA, biological stability

## Abstract

Recent advances in polymerase engineering have made it possible to isolate aptamers from libraries of synthetic genetic polymers (XNAs) with backbone structures that are distinct from those found in nature. However, nearly all of the XNA aptamers produced thus far have been generated against protein targets, raising significant questions about the ability of XNA aptamers to recognize small molecule targets. Here, we report the evolution of an ATP-binding aptamer composed entirely of α-L-threose nucleic acid (TNA). A chemically synthesized version of the best aptamer sequence shows high affinity to ATP and strong specificity against other naturally occurring ribonucleotide triphosphates. Unlike its DNA and RNA counterparts that are susceptible to nuclease digestion, the ATP-binding TNA aptamer exhibits high biological stability against hydrolytic enzymes that rapidly degrade DNA and RNA. Based on these findings, we suggest that TNA aptamers could find widespread use as molecular recognition elements in diagnostic and therapeutic applications that require high biological stability.

## 1. Introduction

Aptamers are functional nucleic acid molecules that fold into 3D structures with specific ligand-binding activity [1]. Over the years, aptamers have been generated by in vitro selection to bind a wide range of targets from small molecules to whole cells and viruses [2]. While antibodies have long been considered the gold standard in molecular recognition, aptamers offer several advantages, including their relative ease of manufacturing through solid-phase synthesis, improved scalability with minimal batch-to-batch variability, and their small size and reduced immunogenicity, which allows for greater penetrance and efficacy as therapeutic agents [3]. Moreover, the in vitro selection process used to generate aptamers allows for greater control over the binding conditions, which enables aptamers to be tailored for specific applications [4].

Small molecules play key roles in many biological processes, functioning as toxins, nutrients, enzyme cofactors, sources of energy, and as cell signaling molecules. However, small molecule targets account for only ~23% of aptamers produced by in vitro selection, making them significantly less abundant than protein aptamers [4]. Among the various small molecule targets chosen for aptamer development, none have been more extensively explored than adenosine triphosphate (ATP). The classic ATP-binding RNA aptamer is one of the simplest solutions to the question of how an RNA molecule can fold into a shape that recognizes adenosine, which is why this aptamer has been discovered multiple times in several independent selections targeting ATP and ATP cofactors [5,6,7,8]. In addition, the large number of ATP analogs available to study the cellular role of ATP in biological processes have made it possible to systematically explore the specificity of new ATP-binding aptamers [9,10,11].

Although most aptamers are composed of DNA and RNA, or close structural analogs that are still recognized by natural polymerases, recent advances in the field of polymerase engineering have made it possible to extend the principles of Darwinian evolution to xeno-nucleic acids (XNAs) with novel backbone structures [12]. Compared to natural genetic polymers, XNAs offer greater resistance against nuclease digestion, making XNA aptamers better suited for practical applications that require high biological stability [13]. Other examples of modified aptamers include SOMAmers, which carry function-enhancing base modifications that allow for higher affinity binding through slower off-rate kinetics [14]. SomaLogic, for example, has used this approach to develop an array-based point-of-care technology for monitoring protein levels in human serum [15].

**Figure 1:**
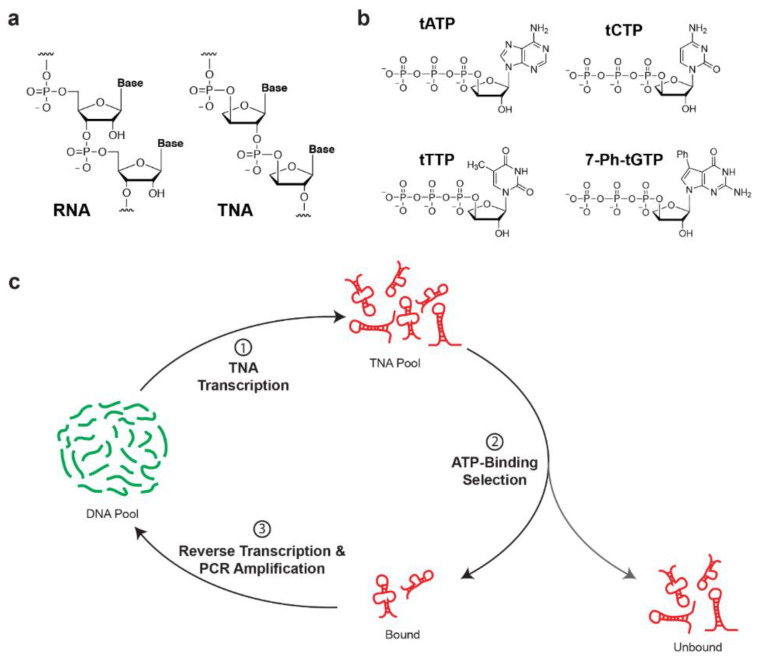
Selection of threose nucleic acid (TNA) aptamers. (**a**) Chemical structures of RNA and TNA. (**b**) tNTP substrates used to enzymatically synthesize TNA molecules for in vitro selection. (**c**) Cartoon image of the in vitro selection strategy used to isolate ATP-binding TNA aptamers.

Threose nucleic acid (TNA, Figure 1) is a type of XNA in which the natural ribose sugar found in RNA has been replaced with an unnatural threose sugar [16]. Despite having a backbone repeat unit that is one atom shorter than the repeat unit found in natural DNA and RNA, TNA is capable of forming antiparallel Watson–Crick duplex structures with DNA, RNA, and itself [17,18]. Although TNA aptamers have been generated against several protein targets [19,20,21], very little is known about the ability for TNA to recognize small molecule targets with high affinity and specificity [22]. Here, we report the in vitro selection of an ATP-binding TNA aptamer that shows high specificity against other nucleotide triphosphates and strong resistance to nuclease degradation. These findings confirm that TNA has the ability to fold into shapes that can recognize small molecule targets with high affinity and high specificity.

## 2. Results and Discussion

### 2.1. Aptamer Selection

TNA molecules with ATP-binding activity were generated by in vitro selection. The selection strategy (Figure 1c) was initiated from a library of ~10^14^ DNA templates, each containing a central random region of 40 nucleotide (nt) positions flanked by fixed-sequence primer binding sites. The single-stranded (ss) DNA library was transcribed into a pool of ssTNA by extending an IR680 labelled DNA primer annealed to the DNA library with TNA triphosphates (Figure 1b). TNA synthesis was performed using a laboratory evolved TNA polymerase known as Kod-RI and a TNA triphosphate mixture that contained the unnatural base analog 7-deaza-7-phenyl guanine in place of the standard guanine base [23]. After purification by denaturing polyacrylamide gel electrophoresis (PAGE), the TNA library was refolded and incubated with ATP-derivatized agarose beads. Non-functional sequences were removed by washing the beads with buffer and functional TNA molecules that remained bound to the ATP-coated beads were recovered by competitive elution using wash buffer that was supplemented with 5 mM ATP. The eluted TNA sequences were reverse-transcribed into cDNA using a natural DNA polymerase isolated from the bacterial species *Geobacillus stearothermophilus* (Bst), which is known to function with general XNA reverse transcriptase activity [24,25]. The reverse-transcribed DNA was then amplified by PCR to produce a population of double-stranded DNA. The ssDNA required to continue the selection was generated in a second PCR step that utilized a PEG-modified PCR primer that allowed for size-separation by denaturing PAGE. The DNA strand carrying the PEG modification was used as the template for the next round of selection.

After four rounds of in vitro selection and amplification, we began discarding the first two elution fractions as a way to promote the enrichment of aptamers with slower off-rates (i.e., tight binding affinity). In addition to monitoring the selection progress by fluorescence-imaging of the tubes containing the flow through, wash, and elution fractions from each round of selection, we also performed a column binding assay on the TNA pool isolated after seven rounds of selection. The column binding results indicate that ~45% of library bound to and eluted from the ATP-agarose beads, demonstrating that the selection had successfully enriched for a population of TNA aptamers with affinity to ATP. An additional three rounds of selective amplification were performed to favor the enrichment of aptamers from the random region by including short DNA oligonucleotides that were complementary to both primer-binding sites.

### 2.2. Aptamer Screening and Characterization

**Figure 2:**
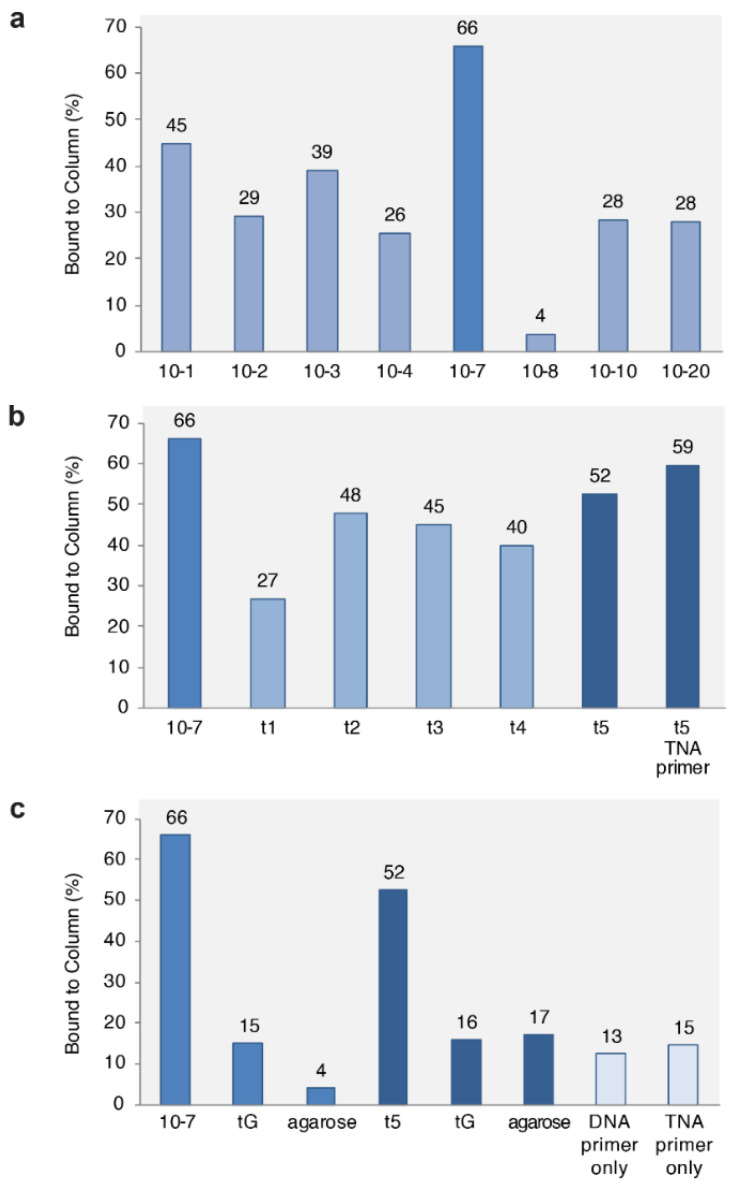
ATP binding activity of in vitro selected TNA aptamers. TNA aptamers tagged with an IR680 dye were evaluated for their ability bind to and elute from ATP-derivatized agarose beads. (**a**) Activity screen of eight unique ATP binding aptamers isolated after 10 rounds of in vitro selection and amplification. (**b**) Truncation analysis of aptamer 10-7 using the technique of 5′ and 3′ end-mapping deletion analysis. (**c**) Binding controls evaluating the contribution of the 7-phenyl-guanine base to the binding activity of the full-length (10-7) and truncated (t5) versions of the TNA aptamer. Other controls included binding assays to underivatized agarose beads as well as the DNA and TNA primer only sequences to ATP derivatized agarose.

The cDNA recovered from round 10 was subjected to next-generation sequencing (NGS) analysis on an Illumina MiSeq platform. Bioinformatic analysis revealed that the top 200 most abundant sequences clustered into multiple sequence families. Based on abundance, enrichment, and sequence family representation, eight sequences were chosen for empirical validation in a column binding assay. The sequences were prepared by enzymatic synthesis using an IR680-labelled primer and DNA templates that were complementary in sequence to the desired TNA molecules. After purification and refolding, the sequences were evaluated for activity in a column-binding assay on ATP-agarose beads. The bound ratio was calculated from the fluorescence intensity of the elution fractions as compared to the flow through and wash fractions using a LI-COR Odyssey imager. Of the eight TNA aptamers tested, seven showed binding activity in the 25–65% range, which is comparable to the round seven pool (Figure 2a). Interestingly, clone T10-8, which differs from clone T10-3 by a single A-to-G mutation abrogates ATP binding activity. Clone T10-7 showed highest binding activity (~65%) and was chosen for further characterization.

End-mapping deletion analysis was used to identify the core ATP binding motif present in clone T10-7. A series of truncations were produced by primer extension using DNA templates that were progressively shortened the 2′ end of the TNA molecule, noting that TNA has a strand polarity of 3′-2′ due to its unique backbone structure (Figure 1a). ATP column-binding assays (Figure 2b) performed on the truncated variants reveals that the T10-7.t5 is the shortest variant with maximum ATP binding activity. This version of the aptamer has a length of 36 nt and exhibits comparable binding activity to the full-length parent sequence (52% versus 66% column binding activity). No significant difference in ATP binding activity was observed when the T10-7.t5 sequence was synthesized with TNA primer in place of the DNA primer used for screening, demonstrating that the all-TNA version of the molecule remains functional (Figure 2b). Control experiments confirm that the 7-phenyl-7-deaza-G residue is important for binding (Figure 2c), as binding activity is significantly reduced when the modified base is replaced with the natural guanine base. Likewise, neither the full-length nor the truncated 10-7 aptamers have affinity for unmodified agarose beads, showing that column binding activity was due to the presence of ATP on the bead surface (Figure 2c). Finally, neither the DNA nor the TNA primers showed any significant activity for the unmodified resin, indicating that the binding activity is due to the evolved sequence rather than the fixed-sequence primer region (Figure 2c).

### 2.3. Binding Affinity and Specificity

**Scheme 1:**
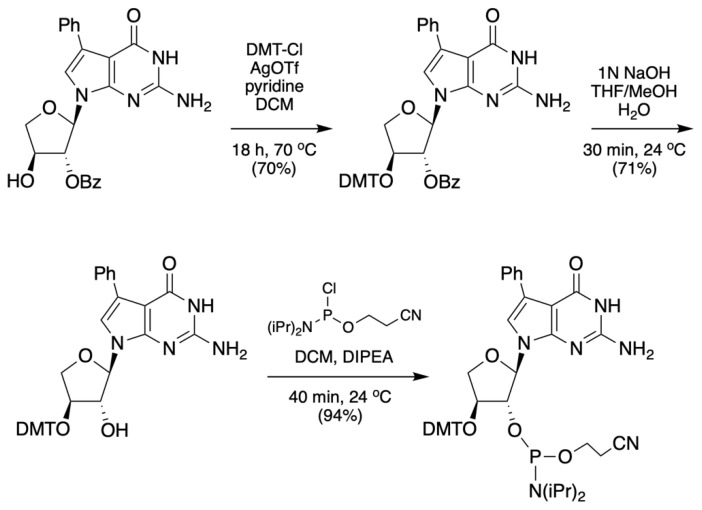
Chemical Synthesis of 7-deaza-7-phenyl guanosine TNA phosphoramidite monomer used for solid-phase synthesis.

**Figure 3:**
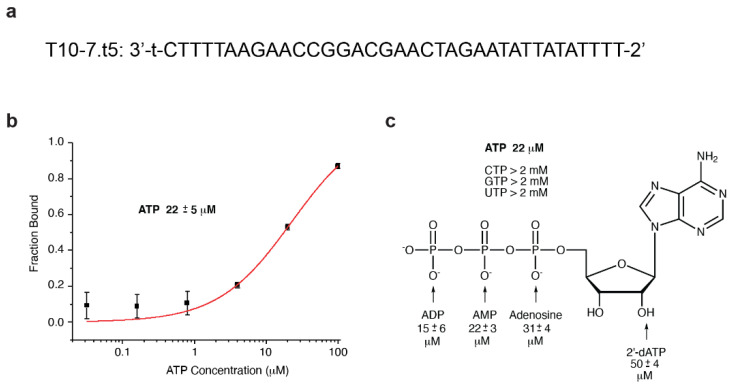
Solution binding affinity (Kd) of the ATP-binding TNA aptamer to free ATP and other closely related nucleoside triphosphates. All binding assays were performed using the minimal sequence (10-7.t5) TNA aptamer prepared by solid-phase synthesis. Binding measurements were performed in binding buffer containing 5 mM MgCl_2_, 300 mM NaCl, and 20 mM Tris-HCl (pH 7.6) at 24 °C. (**a**) Aptamer sequence. (**b**) Binding isotherm obtained for ATP. (**c**) Summary of Kd values obtained for other nucleoside triphosphates and ATP analogs.

We measured the solution binding affinity constant (Kd) of the TNA aptamer T10-7.t5 for free ATP by equilibrium filtration [10]. Since the amount of oligonucleotide required for this technique is not easily achieved by enzymatic synthesis, we decided to generate the truncated aptamer by solid-phase synthesis using TNA phosphoramidite chemistry [26]. The TNA amidites for the natural bases were prepared using previously established methodology [27], while the 7-phenyl-7-deaza-tG phosphoramidite monomer was prepared (Scheme 1) using a slightly modified version of the strategy that was established previously for the triphosphate version of the 7-phenyl-7-deaza-tG monomer [20]. The synthetic oligonucleotide obtained for T10-7.t5 yields a binding isotherm with a K_D_ value of 22 ± 5 μM (Figure 3a), which is comparable to DNA aptamers that were evolved previously to bind ATP [9].

Competitive elution experiments performed against ATP analogs and other nucleoside triphosphates reveal that T10-7.t5 is highly specific for ATP (Figure 3b), with no detectable binding observed for CTP, UTP, or GTP. Close analysis of the affinity measurements performed on ATP analogs reveal that ATP binding is predominantly driven by recognition of the nucleobase, which is similar to what has been observed in other ATP binding aptamers and artificial proteins [5,10].

### 2.4. Biostability

**Figure 4:**
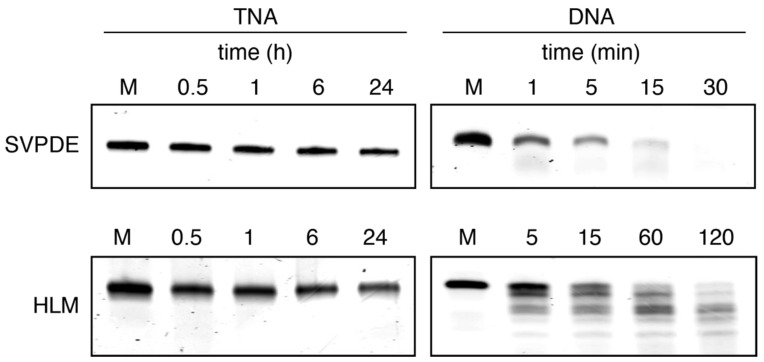
Biological stability assays. Time-dependent assays were performed using snake venom phoshpodiesterase (SVPDE) and human liver microsome (HLM) to evaluate the biological stability of ATP-binding TNA and DNA aptamers. M is a marker for the 0-time point with no enzyme.

One of the advantages of XNA aptamers relative to DNA and RNA aptamers is their enhanced stability against biological nucleases [4]. To evaluate the biological stability of our newly evolved ATP-binding TNA aptamer, we performed a series of digestion assays that compared the stability of T10-7.t5 to a known DNA aptamer (DH25.42) [9]. For this assay, we evaluated the stability of the T10-7.t5 and DH25.42 in concentrated human liver microsomes (HLM), which is considered a rigorous test of oligonucleotide stability due to the abundance and diversity of nucleases present in the media [28]. In addition, we also evaluated the stability of each aptamer against snake venom phosphodiesterase (SVPDE), which is an aggressive enzyme with strong 3′-exonuclease activity commonly employed to evaluate the stability of oligonucleotide therapeutics [29]. The results (Figure 4) clearly show that the TNA aptamer remains undigested after 24 h of incubation under all conditions tested. By comparison, the DNA aptamer is almost completely degraded after 15 min under either nuclease condition. These data confirm earlier findings that TNA is recalcitrant to nuclease digestion [13].

## 3. Conclusions

In conclusion, we demonstrate the in vitro selection of an ATP-binding aptamer composed entirely of threose nucleic acid. The highest affinity aptamer carried a modified 7-phenyl-guanosine residue that was shown to be important for binding. A chemically synthesized version of the aptamer was found to bind ATP with Kd value of ~20 μM, which is similar to known ATP-binding DNA aptamers. However, unlike previous DNA aptamers, the new TNA aptamer is recalcitrant to nuclease digestion. Based on these findings, we suggest that TNA aptamers could find widespread use as molecular recognition elements in diagnostic and therapeutic applications that require high biological stability.

## 4. Materials and Methods

### 4.1. General Information

All chemicals and solvents were purchased from MilliporeSigma (Burlington, MA, USA) and used without further purification. C-8 linked ATP agarose was purchased from MilliporeSigma (Burlington, MA, USA). IR680 dye was purchased from LI-COR (Lincoln, NE, USA). Taq DNA polymerase and 10X ThermoPol buffer were purchased from New England Biolabs (NEB, Ipswich, MA, USA). Kod-RI TNA polymerase and Bst-LF DNA polymerase were expressed and purified as described previously [30,31]. TNA triphosphates bearing natural bases and 7-phenyl-7-deaza-guanine were synthesized as previously described [20,27,32]. DNA oligonucleotides were purchased from Integrated DNA Technologies (Coralville, IA, USA), purified by denaturing polyacrylamide gel electrophoresis (PAGE), electroeluted, buffer exchanged and concentrated using Millipore YM-10 or YM-30 Centricon centrifugal devices, and quantified by UV absorbance. Sequences were cloned into TOPO vectors using TA Cloning Kit, with pCR 2.1 Vector (Thermo Fisher Scientific, Waltham, MA, USA).

### 4.2. TNA Library Preparation

For the aptamer selection, a DNA library was purchased from the Keck Oligonucleotide Synthesis Facility at Yale University. The library contained a 40-mer random region that was flanked on both sides by fixed primer-binding sites (5′-GGATCGTCAGTGCATTGAGATCATTC-(N)_40_-GTTCGTCCGGTTCTTAAAAG-3′, N = A:T:C:G = 35:35:15:15) that were compatible with PBS7 (5′-TCAGTGGGATCGCATTGAGACATTTT-3′) and PBS9 (5′-CTTTTAAGAACCGGACGAAC-3′) forward and reverse DNA primer sequences, respectively. The DNA library was PAGE purified, electroeluted, desalted, and UV quantified. The synthetic library (1 µM) was transcribed into TNA by extending an IR680-labeled PBS9 primer annealed to the DNA library with Kod-RI TNA polymerase (1 µM) and tNTP substrates in ThermoPol buffer (20 mM Tris-HCl, 10 mM (NH_4_)_2_SO_4_, 10 mM KCl, 2 mM MgSO_4_, 0.1% Triton X-100, pH 8.8, NEB) supplemented with 1 mM MnCl_2_ for 3 h at 55 °C. The tNTPs were poised at 100 µM concentration, except for 7-deaza-7-phenyl-tG, which was poised at 75 µM. The pool of TNA molecules was purified by denaturing PAGE, electro-eluted, desalted using Millipore YM-10 microcentrifugal concentrators, and quantified by UV spectroscopy.

### 4.3. Aptamer Selection

ATP agarose columns (1 mL for first round, 0.25 mL for all subsequent rounds) were pre-equilibrated with 10 mL of binding buffer (5 mM MgCl_2_, 300 mM NaCl, and 20 mM Tris-HCl (pH 7.6) at 24 °C). The single-stranded TNA pool (100–250 pmol, 1 mL) in binding buffer was heated to 95 °C for 5 min, cooled to 24 °C for 30 min, and directly loaded onto the ATP-agarose column. For rounds 7–10, 1.1 equivalents of PBS7 and PBS9_rev were added to block the primer binding sites. After equilibrating for 30 min with rotation at 24 °C, the flow through was collected and the column was washed five times with 1 mL of binding buffer. The remaining TNA was specifically eluted five times with 0.25 mL of elution buffer (binding buffer supplemented with 5 mM ATP). The eluate was desalted by exchanging into water and concentrated using Millipore YM-30 microcentrifuge concentrators. The eluate was then reverse transcribed into cDNA using Bst-LF in 20 µL of ThermoPol buffer containing 1 µM of reverse transcription primer PBS7, 500 µM of dNTPs, and 3 mM of supplemental MgCl_2_ at 50 °C for 3 h. The reaction was treated with 0.8 U proteinase K at 50 °C for 20 min, then held at 95 °C for 10 min to denature the enzyme. The cDNA was amplified by PCR using Taq DNA polymerase with PBS7/PBS9 primer pair: 95 °C for 5 min, N cycles of (95 °C for 15 s; 58 °C for 15 s; 72 °C for 30 s). The number of cycles was optimized for each round of selection by sampling PCR reactions every other cycle up to 20 cycles. The amplified DNA was used as template for a second PCR reaction in which PBS7 was replaced with PEGylated PBS7 (PEG-PBS7). The double-stranded pool of enriched DNA templates was separated into single-stranded templates by denaturing PAGE, and the PEGylated strand was used as the DNA template for the next round of selection.

### 4.4. Sequencing

Round 7 and Round 10 cDNA amplicons were generated by PCR using Taq DNA polymerase. Polyclonal cDNA amplicons were purified by 2% agarose using the Monarch DNA Gel Extraction Kit (NEB). The cDNA amplicons were then made into barcoded Illumina libraries using the Apollo 324 platform and PrepX ILM DNA Kit and protocol (Wafergen Bio-Systems, Fremont, CA, USA). The barcoded libraries were spiked into a multiplex of Illumina libraries. The multiplex was denatured and clustered at 12 pM for sequencing on a HiSeq 2500 (Illumina, San Diego, CA, USA) in rapid run mode (8 million reads per amplicon library, single-end 100 cycles) by UCI Genomics High Throughput Facility. Data were analyzed using in-house scripts, and sequences were ordered by abundance.

### 4.5. Binding Assays

TNA aptamers evaluated for ATP binding activity were enzymatically synthesized by extending an IR680-labelled PBS9 primer annealed to a DNA template with TNA. Primer-extensions were performed using Kod-RI TNA polymerase (1 µM) and tNTP substrates in ThermoPol buffer (20 mM Tris-HCl, 10 mM (NH_4_)_2_SO_4_, 10 mM KCl, 2 mM MgSO_4_, 0.1% Triton X-100, pH 8.8, NEB) for 3 h at 55 °C. The tNTPs were poised at 100 µM concentration, except for 7-deaza-7-phenyl-tG, which was poised at 75 µM. The transcribed TNA strands were purified by denaturing PAGE, electro-eluted, desalted using Millipore YM-10 microcentrifugal concentrators, and quantified by UV spectroscopy.

ATP-column binding assays were performed using 0.25 mL of ATP-derivatized agarose beads that were pre-equilibrated with 10 mL of binding buffer. IR680-labelled TNA aptamers were annealed by heated to 95 °C for 5 min, cooled to room temperature for 30 min, and loaded onto the column. After equilibration for 30 min with rotation, the column was drained, and washed five times with 1 mL of binding buffer. The remaining TNA was specifically eluted five times with 0.25 mL of elution buffer (binding buffer supplemented with 5 mM ATP). The fluorescent intensity of each fraction was quantified using LI-COR Odyssey CLx imager.

### 4.6. TNA Oligonucleotide Solid Phase Synthesis and Preparation

TNA phosphoramidites with standard bases were synthesized as previously described [26]. The 7-deaza-7-phenyl-tG phosphoramidite was synthesized based on a previously established protocol for the synthesis of 7-deaza-7-phenyl tGTP monomer [20]. Standard β-cyanoethyl phosphoramidite chemistry and an Applied Biosystems 3400 DNA Synthesizer were used to synthesize TNA oligonucleotides on Universal Support II CPG columns (1 μM scale, Glen Research, Sterling, VA, USA). Standard DNA coupling procedures were modified such that coupling time for TNA amidites is increased to 2000 s and detritylation is performed in two cycles, 60 s each. Cleavage from the solid support was achieved in NH_4_OH (33%) for 18 h at 55 °C. Oligonucleotides were precipitated with butanol and purified by 10% denaturing PAGE, electroeluted, and desalted.

### 4.7. Binding Affinity Measurement

Solution binding affinity constants (Kd) were determined by equilibrium filtration [33]. The solution (100 μL) of the ligand (0.5 nM, γ-^32^P-ATP) and the aptamer (spanning a concentration of 0.032–100 μM) in binding buffer was incubated for 15 min at 4 °C prior to loading in Microcon YM-10 unit. The solution was centrifuged at 13,000× *g* for 12 s to saturate the membrane, and the filtrate was transferred back to the unit. The solution was centrifuged for another 20 s, and the filtrate (~20 μL) was collected. Aliquots (10 μL) were taken from both the remaining solution and the filtrate, and radioactivity in each aliquot was quantified by scintillation counting. The concentration of the aptamer-bound ligand was calculated from the difference between the ligand in the filtrate and in the remaining solution. The apparent Kd values were calculated based on Hill plot fitting using Origin [34]. The binding affinity constants of ATP analogs was measured by displacing bound γ-^32^P -ATP from the aptamers by increasing concentration of unlabeled competitors. The reactions in binding buffer were incubated for 30 min at 4 °C prior to loading in Microcon YM-10 unit. The solution was centrifuged at 13,000× *g* for 12 s to saturate the membrane, and the filtrate was transferred back to the unit. The solution was centrifuged for another 20 s, and the filtrate (~20 μL) was collected. Aliquots (10 μL) were taken from both the remaining solution and the filtrate, and radioactivity in each aliquot was quantitated by scintillation counting. The percent of total radio-labelled ATP bound to the aptamer was then calculated and plotted against the unlabeled competitor concentration.

### 4.8. Nuclease Challenge

Folded IR680-labeled aptamers (TNA T10-7 and DNA DH25.42) at a concentration of 10 nM were incubated at 37 °C for up to 24 h with 1 mg/mL human liver microsome (Sekisui XenoTech, Kansas City, KS, USA) in aptamer folding buffer (20 mM Tris-HCl pH 7.4, 140 mM KCl, 5 mM NaCl, 1 mM MgCl_2_, 1 mM CaCl_2_, 0.005% *v*/*v* Tween 20) or 0.01 mU/μL of snake venom phosphodiesterase (Sigma-Aldrich, St. Louis, MO, USA) in 40 mM Tris-HCl pH 8.4 and 10 mM MgCl_2_. Aliquots of reaction were quenched with formamide and EDTA, and the samples were analyzed for degradation by 20% denaturing PAGE using LI-COR Odyssey CLx imager.
